# Supplementary material for: A SWI/SNF-dependent transcriptional regulation mediated by POU2AF2/C11orf53 at enhancer
Source: Nat Commun. 2024 Mar 7;15:2067. doi: 10.1038/s41467-024-46492-5 (PMC10920751; doi:10.1038/s41467-024-46492-5)
Supplement: Supplementary file 1 — Supplementary Information [file 41467_2024_46492_MOESM1_ESM.pdf]

A SWI/SNF-dependent transcriptional regulation mediated by POU2AF2/C11orf53 at enhancer

Aileen Patricia Szczepanski[1, 2, #], Natsumi Tsuboyama[1, 2, #], Huijue Lyu[1, 2, #], Ping Wang[1, 2], Oguzhan Beytullahoglu[1, 2], Te Zhang[1, 2], Benjamin D Singer[1, 2, 3], Feng Yue[1, 2, 4], Zibo Zhao[1, 2, *], Lu Wang[1, 2, 5, *]

[1]Department of Biochemistry and Molecular Genetics, Feinberg School of Medicine, Northwestern University, Chicago, IL 60611. [2]Simpson Querrey Center for Epigenetics, Feinberg School of Medicine, Northwestern University, Chicago, IL 60611. [3]Division of Pulmonary and Critical Care Medicine, Department of Medicine, Feinberg School of Medicine, Northwestern University, Chicago, IL 60611. [4]Robert H. Lurie Comprehensive Cancer Center, Feinberg School of Medicine, Northwestern University, Chicago, IL, USA

[#]A.P. Szczepanski, N. Tsuboyama, and H. Lyu contributed equally to this article

*Correspondence: zibo.zhao@northwestern.edu (Z.Z.), lu.wang1@northwestern.edu (W.L.)

[5] Lead Contact: Lu Wang

Department of Biochemistry and Molecular Genetics

Simpson Querrey Center for Epigenetics

Northwestern University Feinberg School of Medicine

SQBRC 7-404, 303 E. Superior St.

Chicago, IL 60611

Competing Interests: The authors declare that they have no competing interests.

Supplementary Information

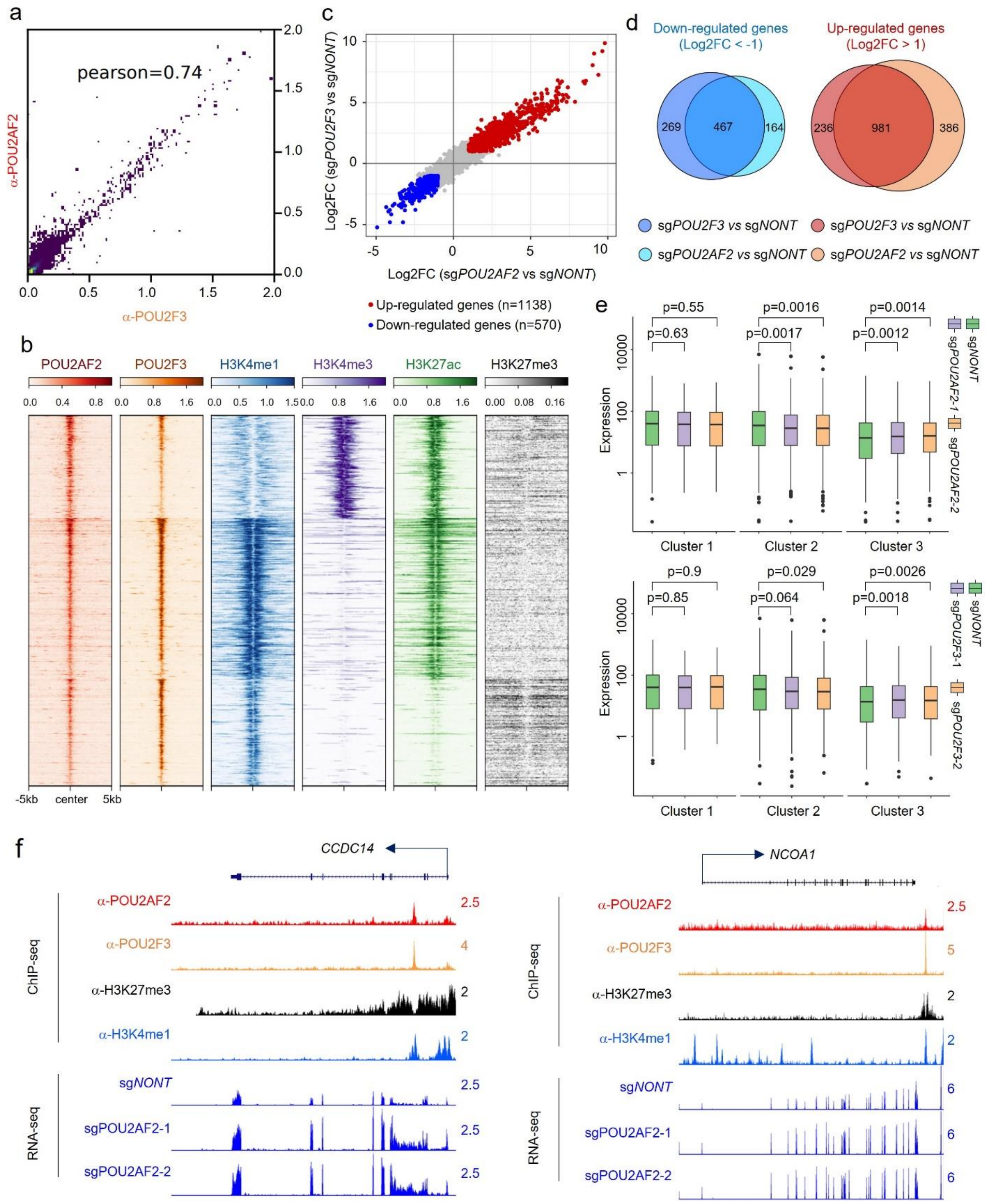

Supplementary Figure 1. POU2AF2 elicits opposing effects of gene expression at distal enhancer elements.

A) The scatter plot shows the correlation between POU2F3 and POU2AF2 peaks in NCI-H526 cells. Pearson correlation = 0.74. B) The overlapped POU2AF2 and POU2F3 peaks in NCI-H211 cells were divided into three clusters by k-means clustering based on POU2AF2, POU2F3, and histone marks (H3K4me1, H3K27ac, H3K4me3, and H3K27me3). The ChIP-seq signal of these histone marks were further centered on the three clusters. C) The scatter plot shows the correlation of gene expression change when POU2F3 or POU2AF2 were depleted by sgRNAs in NCI-H211 cells. The significantly altered genes ($|log2FC| > 1$, adj.p < 0.01) by POU2F3/POU2AF2 depletion were highlighted in red (upregulated, n = 1138) or blue (downregulated, n = 570). Data are derived from two biological replicates. Genes with Benjamini-Hochburg adjusted p-values less than 0.01 were considered to be differentially expressed in the EdgeR analysis[49]. D) The Venn diagram shows the overlap between POU2F3 and POU2AF2 target genes in NCI-H211 cells. E) The box plot shows the significance of expression change of each cluster nearby genes upon the loss of POU2AF2 (upper panel) or POU2F3 (lower panel) with sgRNAs. p-value is calculated by two-sided Wilcoxon test. Center line: median; top and bottom hinges of box: the third and first quantiles; whiskers: quartiles $\pm$ 1.5 $\times$ interquartile range. F) The track examples show the occupancy of POU2F3 and POU2AF2 at H3K27me3 and H3K4me1 occupied enhancer regions in NCI-H211 cells, and the activation of nearby gene expression upon the loss of POU2AF2.

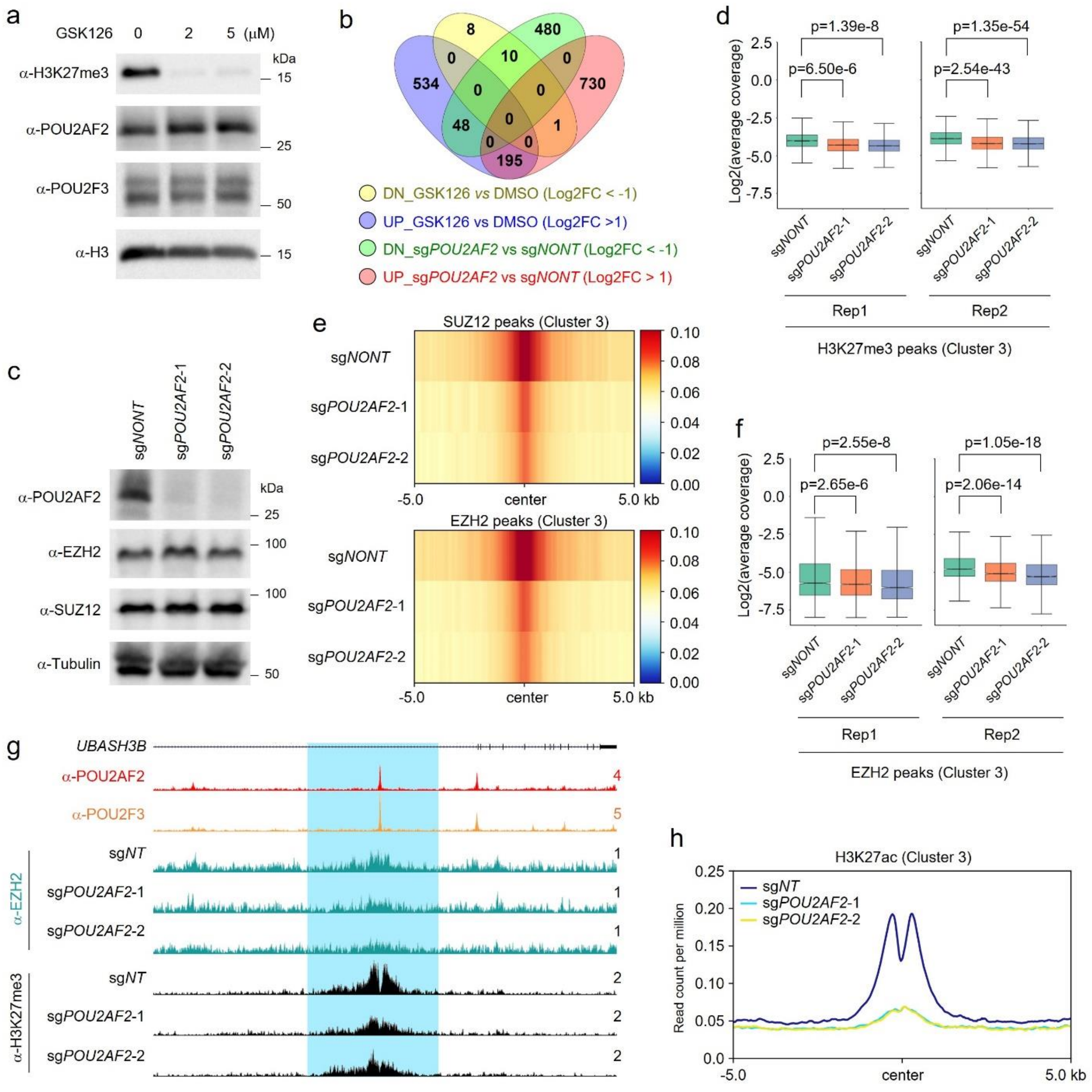

Figure S2. POU2AF2 is essential for PRC2 maintenance and repression of Polycomb target genes.

A) NCI-H526 cells were treated with various concentrations of EZH2 inhibitor GSK126 for 6 days, and the protein levels of H3K27me3, POU2AF2, and POU2F3 were determined by western blot. The total histone H3 was used as internal control. $n = 2$ biologically independent experiments.

Source data are provided as a Source Data file. B) The Venn diagram shows the overlap of the significantly differentially expressed genes in GSK126 treated or POU2AF2 depleted cells of Figure 2a (adj.p < 0.01, $|log2FC| > 1$). Data are derived from two biological replicates. NCI-H526 cells were transduced with two distinct POU2AF2 sgRNAs. C) The protein levels of POU2AF2, EZH2, and SUZ12 were determined by western blot. The total histone H3 was used as internal control. $n = 2$ biologically independent experiments. Source data are provided as a Source Data file. D) The log2FC box plot shows the H3K27me3 levels at Cluster 3 loci in cells transduced with either non-targeting sgRNA or two distinct POU2AF2 sgRNAs. $n = 2$ biologically independent experiments. p-value is calculated by two-sided Wilcoxon test. Center line: median; top and bottom hinges of box: the third and first quantiles; whiskers: quartiles $\pm$ 1.5 $\times$ interquartile range. E) The bar heatmap plots show the chromatin occupied SUZ12 and EZH2 levels at cluster 3 regions. F) The log2FC box plot shows the EZH2 levels at Cluster 3 loci in cells transduced with either non-targeting sgRNA or two distinct POU2AF2 sgRNAs. p-value is calculated by two-sided Wilcoxon test. Center line: median; top and bottom hinges of box: the third and first quantiles; whiskers: quartiles $\pm$ 1.5 $\times$ interquartile range. G) The track example shows the reduction of EZH2 and H3K27me3 levels at *UBASH3B* gene locus with POU2AF2 depletion. $n = 2$ biologically independent experiments. H) The average plots show the H3K27ac levels in POU2AF2 wild type and depleted cells at Cluster 3 regions.

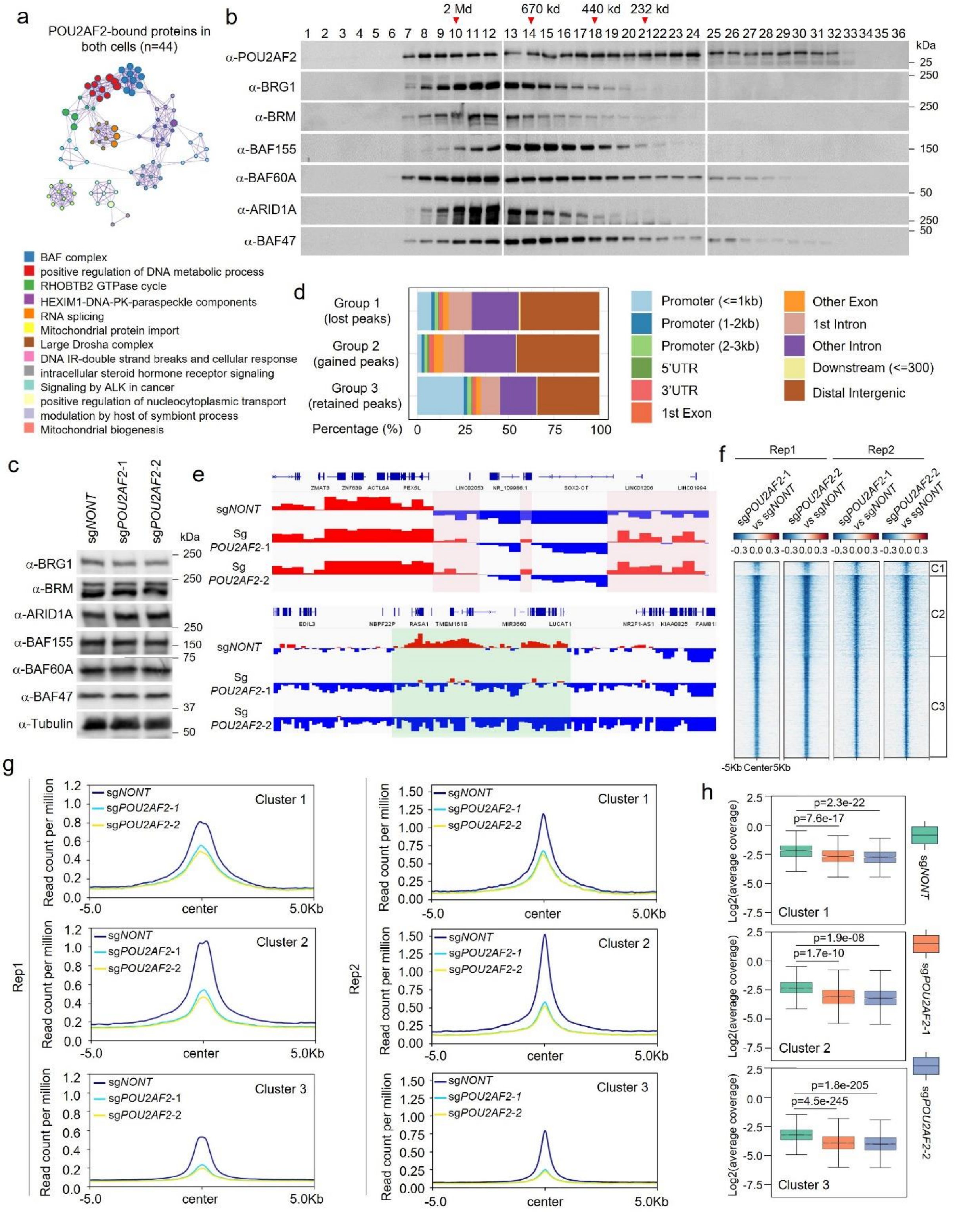

Figure S3. POU2AF2 interacts with the SWI/SNF complex and regulates chromatin accessibility.

A) The enrichment analysis of POU2AF2 co-purified proteins in both NCI-H526 cells and HEK293T cells. B) The nuclear extract from NCI-H526 cells was subjected to size exclusion chromatography, and protein levels of POU2AF2, BRG1, BRM, BAF155, BAF60A, ARID1A, and BAF47 were determined by western blot analysis in the eluted fractions. $n = 2$ biologically independent experiments. Source data are provided as a Source Data file. C) NCI-H526 cells were transduced with lentivirus expressing two distinct sgRNAs targeting POU2AF2. The protein levels of BRG1, BRM, BAF250A, BAF155, BAF60A, and BAF47 were determined by western blot. β-tubulin was used as the internal control. $n = 2$ biologically independent experiments. Source data are provided as a Source Data file. D) The bar chart shows the feature distribution of lost, gained, and retained BRG1 in wild type and POU2AF2 depleted cells. E) A/B compartment designations at 100-kb resolution in sg*NONT*, sg*POU2AF2*-1, and sg*POU2AF2*-2 cells. A compartment, red; B compartment, blue. Regions of B to A switch between sg*NONT* and sg*POU2AF2*, light pink; regions of A to B switch, light green. Both A to B and B to A compartmental switches occur with POU2AF2 depletion. The chromatin occupancy of BRG1 was determined by ChIP-seq in NCI-H526 cells transduced with either non-targeting sgRNA or two distinct POU2AF2 sgRNAs. The log2 fold change heatmap (F) and the average plot (G) shows the loss of BRG1 occupancy in POU2AF2 depleted cells. $n = 2$ biologically independent experiments. H) The box plot shows the reduction of BRG1 in each cluster defined in Figure 1a. p-value is calculated by two-sided Wilcoxon test. Center line: median; top and bottom hinges of box: the third and first quantiles; whiskers: quartiles ± 1.5 × interquartile range.

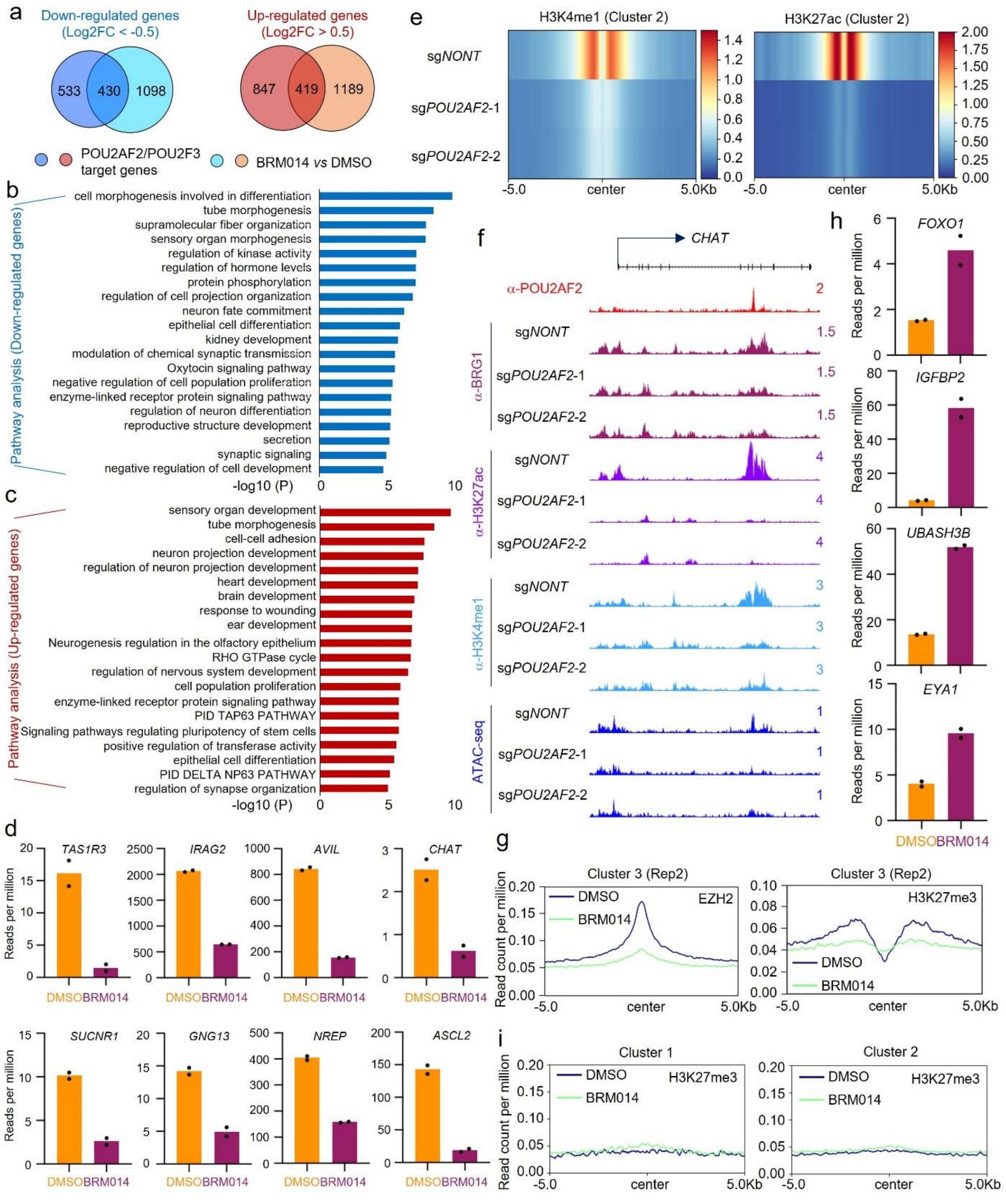

Figure S4. The ATPase activity of SWI/SNF complex is required for POU2AF2 mediated transcriptional regulation.

A) The Venn diagram shows the overlap of the significantly differentially expressed genes in BRG1/BRM inhibitor BRM014 treated or POU2AF2/POU2F3 depleted cells (adj.p < 0.01, |log2FC| > 0.5). Data are derived from two biological replicates. The pathway analysis with the overlapping down-regulated genes (B) and up-regulated genes (C) from (A). The -log10(P) value was calculated by Metascape software[52]. D) The RNA-seq results of the expression levels of *TAS1R3*, *IRAG2*, *AVIL*, *CHAT*, *SUCNR1*, *GNG13*, *NREP*, and A*SCL2* in DMSO or BRM014 (1 μM) treated cells. Data are derived from two biological replicates. Source data are provided as a Source Data file. E) The bar plot heatmap shows the H3K4me1 and H3K27ac levels at Cluster 2 peaks between NCI-H526 cells transduced with either non-targeting sgRNA or two distinct POU2AF2 sgRNAs. F) The track example shows the reduction of BRG1, H3K27ac, H3K4me1 levels as well as ATAC-seq signals at POU2AF2 occupied active enhancer at *CHAT* gene locus. G) The average plot shows a reduction of EZH2 levels (left panel) and H3K27me3 levels (right panel) upon BRM014 treatment at Cluster 3 peaks. H) The RNA-seq results for the expression levels of *FOXO1*, *IGFBP2*, *UBASH3B*, and *EYA1* in DMSO or BRM014 (1 μM) treated cells. Data are derived from two biological replicates. Source data are provided as a Source Data file. I) The average plot shows the H3K27me3 levels at Cluster 1 and Cluster 2 peaks upon BRM014 treatment.

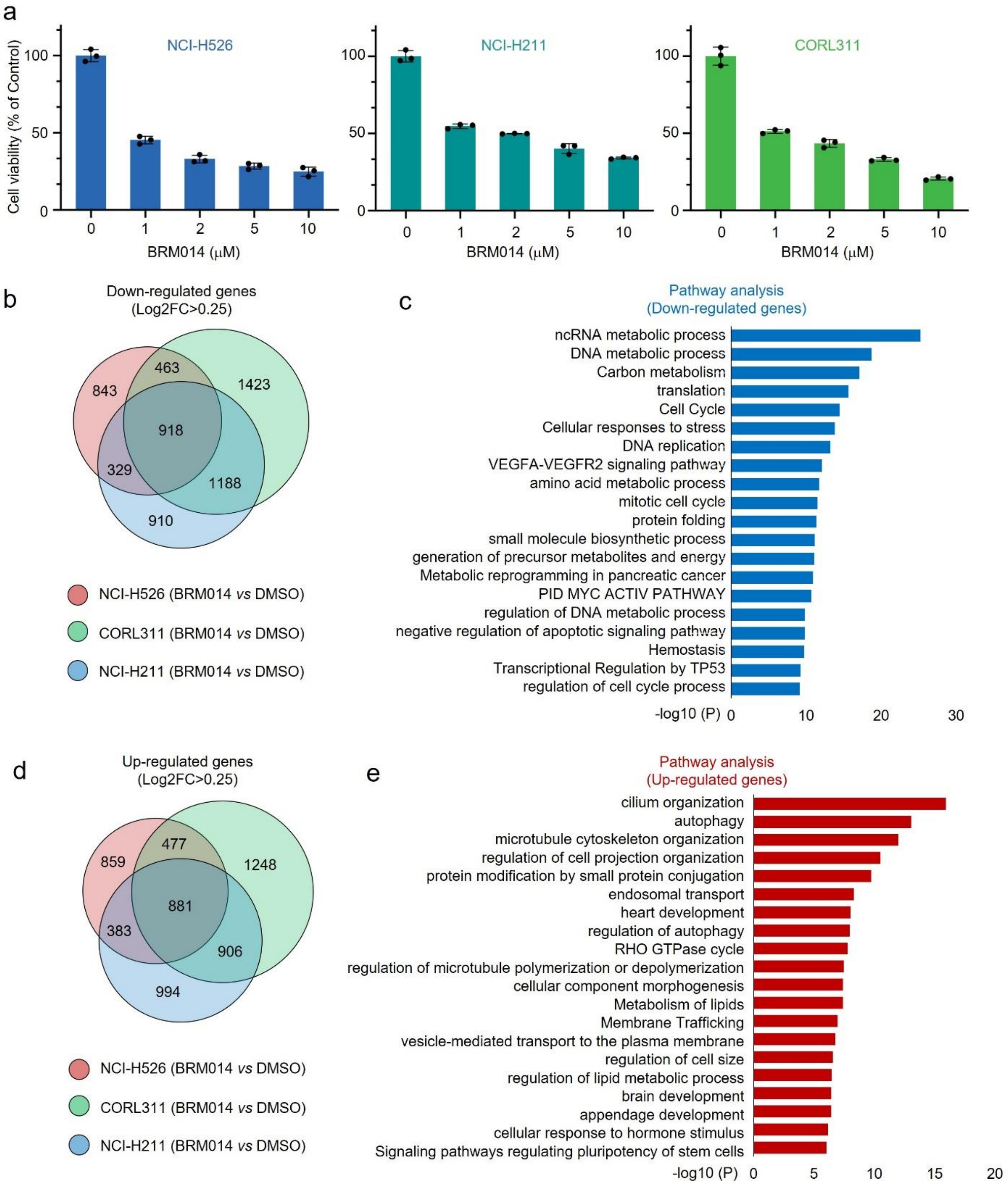

Figure S5. Inhibition of the ATPase activity of SWI/SNF complex as a therapeutic strategy for SCLC treatment.

A) Three different SCLC cell lines NCI-H526, NCI-H211, and CORL311 were treated with different concentrations of BRM014 for 72 hours. The cell viability was determined by CellTiter-Glo Luminescent Cell Viability Assay. $n = 3$ biologically independent experiments. Data are presented as mean values ± standard deviation (SD). Source data are provided as a Source Data file. Three different SCLC cell lines NCI-H526, NCI-H211, and CORL311 were treated with various concentrations of BRM014 for 24 hours. B) The Venn diagram shows the overlap of down-regulated genes in all three cell lines determined by RNA-seq analysis. Data are derived from two biological replicates. C) Pathway analysis of the overlapping down-regulated genes in all the three cell lines treated with BRM014. The -log10(P) value was calculated by Metascape software[52]. D) The Venn diagram shows the overlap of up-regulated genes in all three cell lines determined by RNA-seq analysis. Data are derived from two biological replicates. E) Pathway analysis of the overlapping up-regulated genes in all three cell lines treated with BRM014. The -log10(P) value was calculated by Metascape software[52].
