# Supplementary material for: A SWI/SNF-dependent transcriptional regulation mediated by POU2AF2/C11orf53 at enhancer
Source: Nat Commun. 2024 Mar 7;15:2067. doi: 10.1038/s41467-024-46492-5 (PMC10920751; doi:10.1038/s41467-024-46492-5)
Supplement: Supplementary file 3 — Reporting Summary [file 41467_2024_46492_MOESM3_ESM.pdf]

nature portfolio

Corresponding author(s): Lu Wang

Last updated by author(s): 01/17/2024

Reporting Summary

Nature Portfolio wishes to improve the reproducibility of the work that we publish. This form provides structure for consistency and transparency in reporting. For further information on Nature Portfolio policies, see our Editorial Policies and the Editorial Policy Checklist.

Statistics

For all statistical analyses, confirm that the following items are present in the figure legend, table legend, main text, or Methods section.

n/a	Confirmed	
☐	☒	The exact sample size (*n*) for each experimental group/condition, given as a discrete number and unit of measurement
☒	☐	A statement on whether measurements were taken from distinct samples or whether the same sample was measured repeatedly
☐	☒	The statistical test(s) used AND whether they are one- or two-sided<br>*Only common tests should be described solely by name; describe more complex techniques in the Methods section.*
☒	☐	A description of all covariates tested
☒	☐	A description of any assumptions or corrections, such as tests of normality and adjustment for multiple comparisons
☐	☒	A full description of the statistical parameters including central tendency (e.g. means) or other basic estimates (e.g. regression coefficient) AND variation (e.g. standard deviation) or associated estimates of uncertainty (e.g. confidence intervals)
☐	☒	For null hypothesis testing, the test statistic (e.g. *F*, *t*, *r*) with confidence intervals, effect sizes, degrees of freedom and *P* value noted<br>*Give P values as exact values whenever suitable.*
☒	☐	For Bayesian analysis, information on the choice of priors and Markov chain Monte Carlo settings
☒	☐	For hierarchical and complex designs, identification of the appropriate level for tests and full reporting of outcomes
☒	☐	Estimates of effect sizes (e.g. Cohen's *d*, Pearson's *r*), indicating how they were calculated

Our web collection on statistics for biologists contains articles on many of the points above.

Software and code

Policy information about availability of computer code

Data collection	No software was used to collect open source data
Data analysis	For RNA-seq, gene counts were computed by HTSeq v0.6.1 and used as input for edgeR v3.0.8. Genes with Benjamini-Hochburg adjusted p-values less than 0.01 were considered to be differentially expressed unless otherwise specified. RNA-seq heatmaps adjacent to ChIP-seq heatmaps display log2 (fold change) values of genes corresponding to nearest to ChIP-seq peaks and were displayed using Java TreeView v3.0. GO functional analysis was carried out using Metascape v3.5 with default parameters. For ChIP-seq analysis, all the peaks were called with the MACS software v2.1.0 using default parameters and corresponding input samples. Metaplots and heatmaps were generated using ngsplot v2.63 or deepTools v3.5.1 to display ChIPseq signals. K-means clustering was also generated using ngsplot v2.63. Peak annotation was performed with ChIPseeker v1.38.0 and motif analysis was performed with HOMER v4.11.

For manuscripts utilizing custom algorithms or software that are central to the research but not yet described in published literature, software must be made available to editors and reviewers. We strongly encourage code deposition in a community repository (e.g. GitHub). See the Nature Portfolio guidelines for submitting code & software for further information.

Data

Policy information about availability of data

All manuscripts must include a data availability statement. This statement should provide the following information, where applicable:

- Accession codes, unique identifiers, or web links for publicly available datasets
- A description of any restrictions on data availability
- For clinical datasets or third party data, please ensure that the statement adheres to our policy

The raw and processed next-generation sequencing data generated in this study have been deposited to the Gene Expression Omnibus (GEO) database under the accession number GSE235704 (https://www.ncbi.nlm.nih.gov/geo/query/acc.cgi?acc=GSE235704). The mass spectrometry proteomics data have been deposited to the ProteomeXchange Consortium via the PRIDE partner repository with the dataset identifier PXD043238. All the remaining details and data associated with this study are available within the Methods section and the main text.

Research involving human participants, their data, or biological material

Policy information about studies with human participants or human data. See also policy information about sex, gender (identity/presentation), and sexual orientation and race, ethnicity and racism.

Reporting on sex and gender	n/a
Reporting on race, ethnicity, or other socially relevant groupings	n/a
Population characteristics	n/a
Recruitment	n/a
Ethics oversight	n/a

Note that full information on the approval of the study protocol must also be provided in the manuscript.

Field-specific reporting

Please select the one below that is the best fit for your research. If you are not sure, read the appropriate sections before making your selection.

☒ Life sciences ☐ Behavioural & social sciences ☐ Ecological, evolutionary & environmental sciences

For a reference copy of the document with all sections, see nature.com/documents/nr-reporting-summary-flat.pdf

Life sciences study design

All studies must disclose on these points even when the disclosure is negative.

Sample size	Sample size selection was based on sample availability, sample variation, and was selected to provide sufficient statistical power to discern significant differences and our previous experience. Sample sizes were indicated in the legend of each Figure and Supplementary Figure. No statistical methods were used to predetermine sample sizes.
Data exclusions	None of the data was excluded from the study.
Replication	All attempts at replication were successful. Numbers of attempts of replication are described in the manuscript.
Randomization	Prior to treatment initiation, aged-matched mice were randomized into groups of equal average of tumor burden. Randomization is not relevant to other experiments for this study as cell-based assays require specific conditions depending on treatment.
Blinding	The NGS samples and animal studies were assigned by a technician not aware of the expected results.

Reporting for specific materials, systems and methods

We require information from authors about some types of materials, experimental systems and methods used in many studies. Here, indicate whether each material, system or method listed is relevant to your study. If you are not sure if a list item applies to your research, read the appropriate section before selecting a response.

Materials & experimental systems

n/a	Involved in the study
☐	☒ Antibodies
☐	☒ Eukaryotic cell lines
☒	☐ Palaeontology and archaeology
☐	☒ Animals and other organisms
☒	☐ Clinical data
☒	☐ Dual use research of concern
☒	☐ Plants

Methods

n/a	Involved in the study
☐	☒ ChIP-seq
☒	☐ Flow cytometry
☒	☐ MRI-based neuroimaging

Antibodies

Antibodies used

POU2F3 (#36135), H3K27ac (#8173), H3K4me1 (#5326), H3K4me3 (#9751), H3K27me3 (#9733), Histone H3 (#4499), Cleaved-PARP (#5625), Cleaved Caspase 3 (#9664), SUZ12 (#3737), EZH2 (#5246), BRG1 (#49360), BRM (#11966), ARID1A (#12354), BAF57 (#11956), BAF155 (#11956), BAF60A (#35070), BAF47 (#91735), and PTEN (#9188) antibodies were purchased from Cell Signaling. Tubulin antibody (E7) was purchased from Developmental Studies Hybridoma Bank. HSP90 (sc-7947) antibody was purchased from Santa Cruz. BRG1 (ab110641) and EZH2 (ab191250) antibodies were purchased from Abcam, and were used for ChIP-seq. The POU2AF2 (C11orf53) antibody was produced in rabbits at Pocono Rabbit Farm And Laboratory Inc. by using full-length POU2AF2 recombinant protein as antigen. For all of the western blot experiments, the antibody dilution is 1: 2k. For immunoprecipitation (IP) and chromatin immunoprecipitation (ChIP), 5 g of antibody per reaction was used.

Validation

Anti-POU2F3 antibody (Cell Signaling Technology 36135) has been validated by the vendor in Western blot, and chromatin immunoprecipitation. (https://www.cellsignal.com/products/primary-antibodies/pou2f3-e5n2d-xp-rabbit-mab/36135)

Anti-H3K27ac (Cell Signaling Technology 8173) has been validated by the vendor in Western blot, and chromatin immunoprecipitation. (https://www.cellsignal.com/products/primary-antibodies/acetyl-histone-h3-lys27-d5e4-xp-rabbit-mab/8173)

Anti-H3K4me1 (Cell Signaling Technology 5326) has been validated by the vendor in Western blot, and chromatin immunoprecipitation. https://www.cellsignal.com/products/primary-antibodies/mono-methyl-histone-h3-lys4-d1a9-xp-rabbit-mab/5326.

Anti-H3K4me3 (Cell Signaling Technology 9751) has been validated by the vendor in Western blot, and chromatin immunoprecipitation. (https://www.cellsignal.com/products/primary-antibodies/tri-methyl-histone-h3-lys4-c42d8-rabbit-mab/9751)

Anti-H3K27me3 (Cell Signaling Technology 9733) has been validated by the vendor in Western blot, and chromatin immunoprecipitation. https://www.cellsignal.com/products/primary-antibodies/tri-methyl-histone-h3-lys27-c36b11-rabbit-mab/9733)

Anti-Histone H3 (Cell Signaling Technology 4499) has been validated by the vendor in Western blot. (https://www.cellsignal.com/products/primary-antibodies/histone-h3-d1h2-xp-rabbit-mab/4499)

Anti-Cleaved-PARP (Cell Signaling Technology 5625) has been validated by the vendor in Western blot. (https://www.cellsignal.com/products/primary-antibodies/cleaved-parp-asp214-d64e10-xp-rabbit-mab/5625)

Anti-Cleaved Caspase 3 (Cell Signaling Technology 9664) has been validated by the vendor in Western blot. (https://www.cellsignal.com/products/primary-antibodies/cleaved-caspase-3-asp175-5a1e-rabbit-mab/9664)

Anti-SUZ12 (Cell Signaling Technology 3737) has been validated by the vendor in Western blot, immunoprecipitation, and chromatin immunoprecipitation. (https://www.cellsignal.com/products/primary-antibodies/suz12-d39f6-xp-rabbit-mab/3737)

Anti-EZH2 (Cell Signaling Technology 5246) has been validated by the vendor in Western blot, immunoprecipitation, and chromatin immunoprecipitation. (https://www.cellsignal.com/products/primary-antibodies/ezh2-d2c9-xp-rabbit-mab/5246)

Anti-BRG1 (Cell Signaling Technology 49360) has been validated by the vendor in Western blot, immunoprecipitation, and chromatin immunoprecipitation. (https://www.cellsignal.com/products/primary-antibodies/brg1-d1q7f-rabbit-mab/49360)

Anti-BRM (Cell Signaling Technology 11966) has been validated by the vendor in Western blot, immunoprecipitation, and chromatin immunoprecipitation. (https://www.cellsignal.com/products/primary-antibodies/brm-d9e8b-xp-rabbit-mab/11966)

Anti-ARID1A (Cell Signaling Technology 12354) has been validated by the vendor in Western blot and chromatin immunoprecipitation. (https://www.cellsignal.com/products/primary-antibodies/arid1a-baf250a-d2a8u-rabbit-mab/12354)

Anti-BAF155 (Cell Signaling Technology 11956) has been validated by the vendor in Western blot, immunoprecipitation, and chromatin immunoprecipitation. (https://www.cellsignal.com/products/primary-antibodies/smarcc1-baf155-d7f8s-rabbit-mab/11956)

Anti-BAF57 (Cell Signaling Technology 11956) has been validated by the vendor in Western blot, immunoprecipitation, and chromatin immunoprecipitation. (https://www.cellsignal.com/products/primary-antibodies/smarce1-baf57-e6h5j-rabbit-mab/33360)

Anti-BAF60A (Cell Signaling Technology 35070) has been validated by the vendor in Western blot, immunoprecipitation, and

chromatin immunoprecipitation. (https://www.cellsignal.com/products/primary-antibodies/smarcd1-baf60a-e7w9w-rabbit-mab/35070)

Anti-BAF47 (Cell Signaling Technology 91735) has been validated by the vendor in Western blot, immunoprecipitation, and chromatin immunoprecipitation. (https://www.cellsignal.com/products/primary-antibodies/smarcb1-baf47-d8m1x-rabbit-mab/91735)

Anti-PTEN (Cell Signaling Technology 9188) has been validated by the vendor in Western blot. (https://www.cellsignal.com/products/primary-antibodies/pten-d4-3-xp-rabbit-mab/9188)

Anti-Tubulin antibody (Developmental Studies Hybridoma Bank, E7) has been validated by the vendor in Western blot. (https://dshb.biology.uiowa.edu/E7_2)

Anti-HSP90 (Santa Cruz Biotechnology sc-7947) has been validated by the vendor in Western blot. (https://www.scbt.com/p/hsp-90alpha-beta-antibody-h-114)

Anti-BRG1 (Abcam ab110641) has been validated by the vendor in western blot and chromatin immunoprecipitation. (https://www.abcam.com/products/primary-antibodies/brg1-antibody-epncir111a-ab110641.html)

Anti-EZH2 (Abcam ab191250) has been validated by the vendor in western blot and chromatin immunoprecipitation. (https://www.abcam.com/products/primary-antibodies/kmt6--ezh2-antibody-epr20108-chip-grade-ab191250.html)

The POU2AF2 antibody was produced in rabbits in house by using full-length POU2AF2 recombinant protein as antigen. It has been validated in western blot, immunoprecipitation, and chromatin immunoprecipitation with wild type and knockout cells in our previous studies (PMID: 36197978)

Eukaryotic cell lines

Policy information about cell lines and Sex and Gender in Research

Cell line source(s)	HEK293T cells were purchased from ATCC, and maintained with DMEM (Fisher Scientific, #15013CV) containing 10% FBS (Sigma). The SCLC cell lines NCI-H526, NCI-H211 and CORL311 were obtained from ATCC, and were maintained with ATCC-formulated RPMI-1640 medium containing 10% FBS (Sigma).
Authentication	We periodically authenticate cell lines by Short Tandem Repeat (STR) profiling.
Mycoplasma contamination	All cells were routinely tested and verified to be free of mycoplasma contamination by PCR based methods.
Commonly misidentified lines (See ICLAC register)	No commonly misidentified cell lines were used.

Animals and other research organisms

Policy information about studies involving animals; ARRIVE guidelines recommended for reporting animal research, and Sex and Gender in Research

Laboratory animals	All animal studies were conducted in compliance with the ethical guidelines. Mice were housed 5 per cage and maintained under specific pathogen-free conditions. Twelve hours of light are provided each day and food and water were provided freely. 5- to 6-week-old athymic nude mice were purchased from the Envigo and were used for xenograft experiments.
Wild animals	No wild animals were used in the study.
Reporting on sex	Sexual differences were not considered as a biological variable.
Field-collected samples	No field collected samples were used in the study.
Ethics oversight	All proposed research activities with vertebrate animals are conducted in the IACUC and AAALAC approved Center for Comparative Medicine (CCM) facilities. This study was approved by Northwestern University Institutional Animal Care and Use Committee (Animal Protocol No. IS00013610).

Note that full information on the approval of the study protocol must also be provided in the manuscript.

Plants

Seed stocks: n/a

Novel plant genotypes: n/a

Authentication: n/a

ChIP-seq

Data deposition

☒ Confirm that both raw and final processed data have been deposited in a public database such as GEO.

☒ Confirm that you have deposited or provided access to graph files (e.g. BED files) for the called peaks.

Data access links<br>*May remain private before publication.*	https://www.ncbi.nlm.nih.gov/geo/query/acc.cgi?acc=GSE235704
Files in database submission	*Provide a list of all files available in the database submission.*
Genome browser session<br>(e.g. UCSC)	*Provide a link to an anonymized genome browser session for "Initial submission" and "Revised version" documents only, to enable peer review. Write "no longer applicable" for "Final submission" documents.*

Methodology

Replicates	For RNA-seq, two biological replicates were performed. For ChIP-seq, two biological replicates were performed for statistically analysis, and one replicated for histone marks that were used to define specific chromatin regions. ATAC-seq and Hi-C, one replicate was provided. Also refer to Methods/RNA-seq analysis/ChIP-seq analysis.
Sequencing depth	At least 30 million reads were provided for each sample. Libraries were sequenced with double-end, 150bp reads.
Antibodies	POU2F3 (#36135), H3K27ac (#8173S), H3K4me1 (#5326S), H3K4me3 (#9751), H3K27me3 (#9733), SUZ12 (#3737) antibodies were purchased from Cell Signaling. BRG1 (ab110641) and EZH2 (ab191250) antibodies were purchased from Abcam. The POU2AF2 antibody was produced in rabbit in house by using full-length POU2AF2 recombinant protein as antigen.
Peak calling parameters	All the peaks were called with the MACS v1.4.2 software using default parameters and corresponding input samples.
Data quality	All of the NGS experiments in this study have passed the quality control of the default setting of the software we used.
Software	We used MACS v1.4.2, Metaplots and ngsplot software.
